# Factors associated with head injury among survivors of motorcycle crashes: a case-control study in northern Ghana

**DOI:** 10.11604/pamj.2022.43.73.35900

**Published:** 2022-10-11

**Authors:** Anthony Baffour Appiah, Patricia Akweongo, Samuel Sackey, Martin Tangnaa Morna, Ernest Kenu, Alexis Dun Bo-ib Buunaaim, Samuel Akobour Yaw Debrah, Thomas Kolawole Ojo, Peter Donkor, Charles Mock

**Affiliations:** 1 Ghana Field Epidemiology and Laboratory Training Programme, School of Public Health, University of Ghana, Legon, Accra, Ghana,; 2 Department of Health Policy, Planning and Management, School of Public Health, University of Ghana, Legon, Accra, Ghana,; 3 Department of Surgery, School of Medical Science, University of Cape Coast, Cape Coast, Ghana,; 4 Department of Surgery, Tamale Teaching Hospital, Tamale, Ghana,; 5 Department of Geography and Regional Planning, Faculty of Social Sciences, College of Humanities and Legal Studies, University of Cape Coast, Cape Coast, Ghana,; 6 Department of Surgery, School of Medical Science, Kwame Nkrumah University of Science and Technology, Kumasi, Ghana,; 7 Harborview Injury Prevention and Research Center, Harborview Medical Center, Seattle, USA

**Keywords:** Motorcycle, risk factors, head injury, case-control, survivors

## Abstract

**Introduction:**

the increasing use of motorcycles in northern Ghana is associated with a high incidence of motorcycle crashes and resultant head injuries. This study sought to determine factors associated with head injuries among survivors of motorcycle crashes in northern Ghana.

**Methods:**

a prospective unmatched case-control study was conducted at the Tamale Teaching Hospital (TTH). A total of 326 cases (victims who suffered a head injury with or without other injuries) and 294 controls (persons who suffered various injuries except for head injury) from motorcycle crashes were consecutively sampled at TTH from December 15, 2019, to May 15, 2020. A semi-structured questionnaire was used to interview patients in addition to medical records review. Factors associated with head injury were examined using multivariable logistic regression at p<0.05 and a 95% confidence interval.

**Results:**

the prevalence of head injury was 53.03% among of 660 survivors of motorcycle crashes. The majority of the patients were young males aged 15-44 years. The rate of helmet use was lower in cases (12.88%) than in controls (57.82%) (p<0.001). Factors associated with head injury were not wearing helmet (AOR= 9.80, 95% CI: 6.22, 15.43), male (AOR=1.75, 95% CI: 1.07, 2.85), student (AOR=0.38, 95% CI: 0.16, 0.91), and alcohol use within 24 hours (AOR=0.17, 95% CI: 0.04, 0.70).

**Conclusion:**

non-use of helmet and male gender significantly increased the risk of head injury risk in this study. Alcohol use and being a student were associated with lower odds of head injuries. Motorcycle safety efforts in the study area should emphasize helmet promotion.

## Introduction

Approximately 1.35 million road traffic deaths (RTDs) are recorded globally every year [1]. The rates of road traffic injuries and deaths (RTIDs) are higher in low- and middle-income countries (LMICs) [1,2]. The LMICs of the African region has an annual rate of RTDs of 26.6/100,000 people, the highest for any region and notably higher than the average rate of 5.1/100,000 in high-income European countries [1]. Ghana is ranked 21^st^, out of 45 African countries in terms of RTDs rates, [2]. The increasing use of motorcycles is compounding road safety concerns in Ghana. Currently, motorized 2-and 3-wheelers account for 18% of RTDs in Ghana [2,3]. Their use is especially high in the northern zone of Ghana, which is one of the lowest-income areas in the country [4]. The leading cause of death for motorcyclists is head injury [5,6]. For instance, 92% of traumatic brain injuries are a result of motorcycle crashes in the Northern Region of Ghana [7]. The risk of sustaining a fatal head injury among motorcyclists has been linked to the non-wearing of helmets in many countries [8,9]. The World Health Organization (WHO) adopted the Haddon matrix model to explore the risk factors of motorcycle injuries in three domains (human, vehicle, and environment) [10]. Previous studies also identified risk factors in three basic categories, namely, sociodemographic factors (e.g, age, male, education, riding experience), crash characteristics (e.g, mechanism of injury, helmet use, alcohol and drug use, riding speed, risk-taking behavior), and environmental or road condition (e.g, poor visibility, time of day, wet or slippery roads) [5,9,11]. Evidence of specific risk factors for head injuries in motorcycle crashes in the Ghanaian context is limited. In northern Ghana where the mode of transport is largely by motorcycle, previous studies only determined the rate and reason for non-helmet use [4,12]. As part of efforts to bridge existing knowledge gaps, this study adopted an unmatched case-control study to examine the factors associated with head injuries among survivors of motorcycle crashes (SMCs) in northern Ghana. Specifically, the study addressed the following questions: i) what is the prevalence of head injuries among SMCs reporting to the main referral center in northern Ghana? ii) is there any significant difference in the sociodemographic, crash and environmental characteristics between SMCs who have head injuries and those who do not and; iii) what behavioral, crash and environmental factors are significantly associated with head injuries among SMCs in northern Ghana?

## Methods

**Figure 1:**
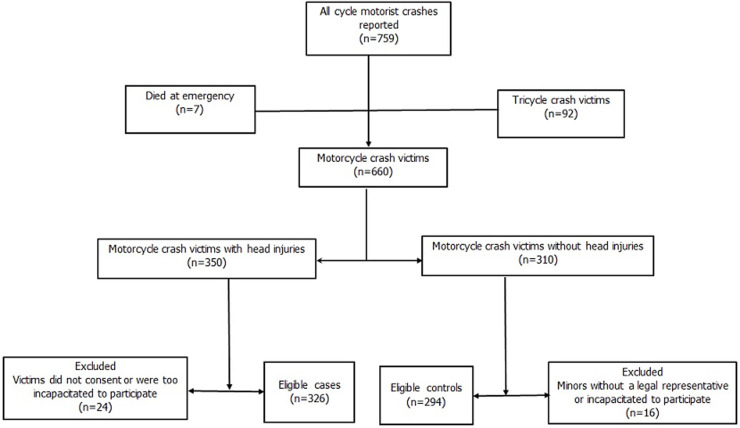
sample selection based on criteria for cases and controls

**Study design and participants:** we conducted a prospective unmatched case-control among the SMCs at the Tamale Teaching Hospital (TTH) in the Northern region of Ghana. The TTH is an 800-bed hospital that serves the Northern half of Ghana and receives cases from parts of neighboring countries such as Burkina Faso and Togo. The study was between December 15, 2019 and May 15, 2020. A patient´s status (case vs. control) was confirmed by a review of their medical records for glagow coma scale (GCS) and abbreviated injury score (AIS). Abbreviated injury score was calculated for the head and other body regions using AIS 2005 [13]. A case was defined as a person who sustained a minor to severe head injury (with or without injuries to other body parts) from a motorcycle crash within two weeks of presentation at the TTH. A control was defined as a person who did not sustain a head injury from motorcycle crashes within two weeks of presentation at the TTH. Head injury was defined as a person diagnosed with GCS < 14 or/and head AIS > 1. A person diagnosed with GCS = 15 and head AIS = 0 were classified as a control (i.e. without head injury). We used OpenEpi (Version 3.03.17) [14] which applied the formula by Kelsey *et al*. [14] aiming to detect an odds' ratio of at least 2.21, assuming a power of 80%, a ratio of cases and controls of 1, and a 95% confidence level. The exposure variable used was not-wearing helmets whose prevalence was estimated at 18% among controls [5]. The sample size calculated was 278. When a 12% non-response rate was accounted for, the desired (minimum) sample size increased to 312 (156 cases and 156 controls). However, a total of 660 SSMCs were reported to TTH over the study period. Both cases and controls were selected consecutively. A summary of sample selection is shown in Figure 1.

**Sources of data and variables:** two sources of data were used in this study - secondary data from medical records and primary data from questionnaires. A semi-structured questionnaire with both open and closed-ended questions was used. The questionnaire had four parts; Part i) patient sociodemographic characteristics (age, gender, marital status, religion, education, occupation and socioeconomic status); Part ii) injury and history, type of injury, abbreviated injury score (AIS) and glasgow coma scale (GCS); Part iii): crash characteristics (including state of involvement, number of riders/users, type of collision and mechanism of injury, riding experience, helmet use, pre-crash speed, alcohol use and mobile phone use), and Part iv): environmental and road conditions (including weather condition, lighting condition, road type, road surface condition, curves, speed limits and visibility of road signs).

**Inclusion and exclusion criteria:** all consecutive SMCs diagnosed and treated at the Accident and emergency unit at TTH from December 15^th^, 2019, to May 15^th^, 2020 were included in the study. Survivors of motorcycle crashes who died within 24 hours of admission, those who were too incapacitated to recount incidents, minors (<16 years) who did not have a legal representative involved in the crash, and people who did not consent were excluded Figure 1.

**Data collection process:** first, informed consent was obtained from all participants who met the inclusion criteria, after a brief description of the purpose, benefits, and potential risks of the study had been explained. Then, the research assistants administered the semi-structured questionnaire to respondents. The medical records were then reviewed to capture diagnosed injuries, radiologic imaging requested, AIS, and GCS.

**Data analysis:** data were analyzed using STATA IC (version 15, StataCorp, USA). We first conducted a bivariate analysis of patients´ sociodemographic, crash, and environmental characteristics to compare the differences between cases and controls with a chi-square test. Logistic regression analysis was used to explore the level of association of specific covariates with the occurrence of head injury. Binary logistic regression models were computed for each sociodemographic, crash and environmental factor. Subsequently, a multivariable regression model was constructed to explore the association between head injury and each risk factor while controlling for the effects of covariates. The criteria for selecting variables into the multivariable model was p< 0.25. The estimated odd ratios (OR) and 95% confidence intervals (CI) were presented in each case. All statistical analyses were considered significant at p<0.05.

**Ethical aprroval:** the study was approved by the Ethics Review Committee of the Ghana Health Service (GHS-ERC024/12/19) and the Department of Research and Development of the Tamale Teaching Hospital (TTH/R&D/SR/154).

## Results

**Sociodemographic characteristics of survivors of motorcycle crash:** there were 660 motorcycle crash victims, including 326 cases and 294 controls. Patients were predominantly males (84.97% male for cases and 72.72% male for controls) with a mean age of 29.35±15.72 years for cases and 29.79±15.43 years for controls. There was no significant difference in age distribution among cases and controls (p=0.294). There were significant differences in the distribution of gender (p<0.001), religion (p=0.001), education (p=0.006), and occupation (p<0.001) between the two groups (Table 1).

**Table 1:** socio-demographic characteristics of patients with head injury involved motorcycle crashes

Variable	Cases	Controls	Chi-square
	N=326 (%)	N =294 (%)	p-value
**Age (years)**			
Mean(SD)	29.35±15.72	29.79±15.43	
**Category**			
<15 years	54 (16.56)	39 (13.27)	0.294
15-44 years	220 (67.48)	218 (74.15)	
45-64 years	41 (12.58)	27 (9.18)	
65 years+	11 (3.37)	10 (3.40)	
**Gender**			<0.001**
Female	49 (15.03)	80 (27.21)	
Male	277 (84.97)	214 (72.79)	
**Marital status**			0.979
Single	156 (47.85)	141 (47.96)	
Married	170 (52.15)	153 (52.04)	
**Religion**			0.001*
Christian	93 (28.53)	54 (18.37)	
Muslim	225 (69.02)	239 (81.29)	
Others	8 (2.45)	1 (0.34)	
**Education**			0.006*
No formal education	117 (35.89)	91 (30.95)	
Primary	102 (31.29)	66 (22.45)	
Junior high school	25 (7.67)	40 (13.61)	
Senior high/technical school	66 (20.25)	74 (25.17)	
Tertiary	16 (4.91)	23 (7.82)	
**Occupation**			<0.001*
Unemployed	90 (27.61)	50 (17.01)	
Formal employment	22 (6.75)	22 (7.48)	
Self-employment	177 (54.29)	154 (52.38)	
Casual worker	18 (5.52)	29 (9.86)	
Student	19 (5.83)	39 (13.27)	
**Socioeconomic status**			0.148
Poorest	33 (10.12)	15 (5.10)	
Poor	78 (23.93)	70 (23.81)	
Middle	150 (46.01)	152 (51.70)	
Rich	51 (15.64)	41 (13.95)	
Richest	14 (4.29)	16 (5.44)	

* Significant at p<0.05 level; **significant at p<0.001 level

**Figure 2:**
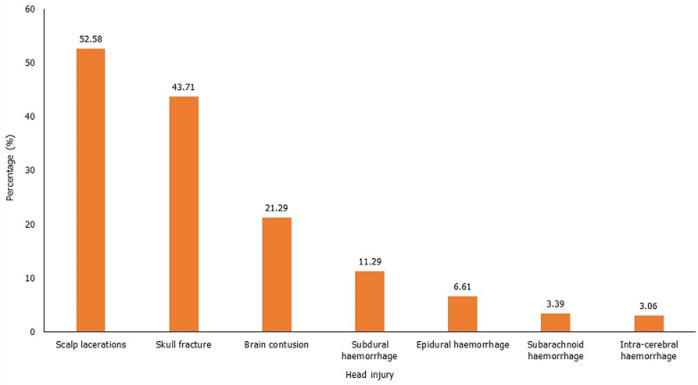
head injuries sustained among survivors of motorcycle crashes in northern Ghana

**Figure 3:**
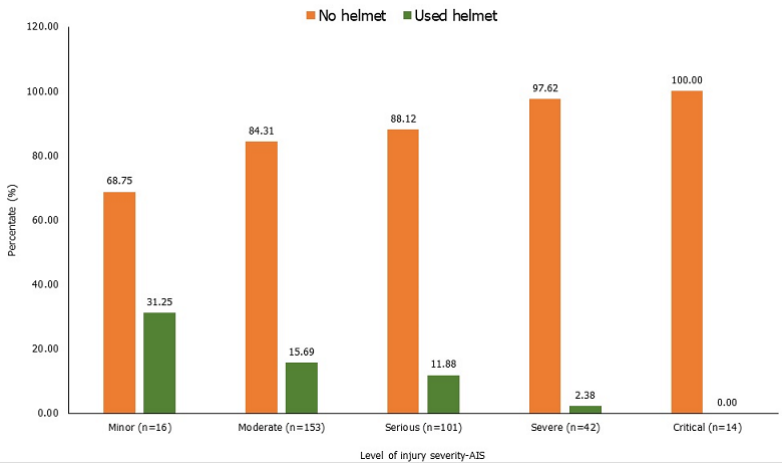
severity of head injuries among survivors of motorcycle crashes in northern Ghana

**Prevalence and severity of head injuries of survivors of motorcycle crash:** the prevalence of head injuries among the 660 motorcycle crash victims was 53.03% (350/660). Figure 2 shows the head and brain lesions sustained by cases. The scalp lacerations (52.58%) and skull fractures (43.71%), and brain contusion (21.29%) were commonly seen in patients (Figure 2). The mean injury severity score (ISS) was higher in cases (16.88¬±9.40) than among controls (12.67± 5.60) (p<0.001). There were no differences in mean length of hospitalization among cases (7.28±8.33 days) and controls (7.57±9.82 days) (p=0.70). The distribution of cases by head AIS and rate of helmet use are shown in Figure 3. The majority of the cases sustained moderate (153, 46.98%) and serious (101, 30.98%) head injuries. Lower rates of helmet use were reported among patients with severe levels of head-AIS (severe (97.62% of all severe head injuries were to patients who were not using helmets) and critical (100%)) (Figure 3).

**Crash characteristics of survivors of motorcycle crashes:** Table 2 summarizes the crash characteristics of patients with head injuries involved in motorcycle crashes. Most of the patients were riders and single occupants. Collision with other motorcycles was the most common type of collision in each group but was more common in controls (45.92%) than in cases (29.14%) (p<0.001). The most common category of riding experience was 6-10 years in each group, but this category was more common among controls (48.64%) than cases (40.18%) (p=0.003). The rate of helmet use was much lower in cases (12.88%) than in controls (57.82%) (p<0.001) (Table 2).

**Table 2:** crash characteristics of patients with head injury involved in motorcycle crashes

Crash characteristics	Cases	Controls	Chi-square
	N=326 (%)	N=294 (%)	p-value
**State of involvement**			0.659
Rider	240 (73.62)	221 (75.17)	
Pillion	86 (26.38)	73 (24.83)	
**Number of occupants**			0.983
occupant	170 (52.15)	154 (52.38)	
occupants	151 (46.32)	136 (46.26)	
occupants+	5 (1.53)	4 (1.36)	
**Collided with**			<0.001**
Road object	53 (16.26)	31 (10.54)	
Another motorcycle	95 (29.14)	135 (45.92)	
Other vehicle	92 (28.22)	54 (18.37)	
Pedestrian	86 (26.38)	74 (25.17)	
**Riding experience**			0.003*
0-5 years	110 (33.74)	107 (36.39)	
6-10 years	131 (40.18)	143 (48.64)	
>10 years	85 (26.07)	44 (14.97)	
**Helmet used**			<0.001**
No helmet	284 (87.12)	124 (42.18)	
Used helmet	42 (12.88)	170 (57.82)	
**Pre-crash speed (**km/hr)			0.870
Under speed limit (≤50)	121 (37.12)	111 (37.76)	
Over speed limit (>50)	205 (62.88)	183 (62.24)	
**Riding under influence of alcohol**			0.018*
Non-alcohol	323 (99.08)	283 (96.26)	
Alcohol influence	3 (0.92)	11 (3.74)	
**Using mobile phones while riding**			0.264
No phone use	317 (97.24)	281 (95.85)	
Phone use	9 (2.76	13 (4.42)	

* Significant at p<0.05 level;**significant at p<0.001 level

**Environmental characteristics of motorcycle crashes:** as shown in Table 3, dry weather, daylight, urban road, concrete with potholes, roads with a speed limit, and visible of road signs were the most common environmental characteristics. Dry weather conditions were more common among cases (97.85%) than in controls (94.56%) (p=0.03). Concrete roads with potholes were more common as road surface conditions for cases (66.87%) than controls (63.27%) (p<0.001). There were also significant differences in presence of curves (p<0.001), the presence of speed limits (p=0.001), and the visibility of road signs (p<0.001) between the two groups (Table 3).

**Table 3:** environmental characteristics of motorcycle crashes in which patients sustained head injury

Environmental characteristics	Cases	Controls	Chi-square
	N=326 (%)	N =294 (%)	p-value
**Weather condition**			0.030*
Wet/slippery	7 (2.15)	16 (5.44)	
Dry	319 (97.85)	278 (94.56)	
**Lighting condition**			0.115
Daylight	186 (57.06)	186 (63.27)	
Reduce light/darkness	140 (42.94)	108 (36.73)	
**Road type**			0.068
Rural road	57 (17.48)	36 (12.24)	
Urban road	269 (82.52)	258 (87.76)	
**Road surface condition**			<0.001**
Untarred/earthen road	55 (16.87)	23 (7.82)	
Concrete with potholes	218 (66.87)	186 (63.27)	
Asphalt	53 (16.26)	85 (28.91)	
**Presence of curves**			<0.001**
No curve	191 (58.59)	114 (38.78)	
Curve	135 (41.41)	180 (61.22)	
**Presence of speed limit**			0.001*
No speed limit	98 (30.06)	55 (18.71)	
Speed limit	228 (69.94)	239 (81.29)	
**Visibility of road sign**			<0.001**
No road sign	108 (33.13)	58 (19.73)	
Road sign	218 (66.87)	236 (80.27)	

* Significant at p<0.05 level; **significant at p<0.001 level

**Analysis of factors associated with head injuries - crude odd ratios:** the crude odd ratios (CORs) for head injury-related factors in motorcycle crashes are shown in Table 4 and Table 5. Male victims had a 2-fold increase in odds of head injury compared to females (COR=2.11, 95% CI: 1.41, 3.15). Non-helmet use (COR=9.27, 95% CI: 6.23, 13.80) and long-time (above 10 years) of riding experience (COR=1.88, 95% CI: 1.20, 2.95) were associated with increased odds of head injury. Alcohol consumption was associated with lower crude OR of 0.24 (95% CI: 0.07, 0.87) (Table 4). All the environmental factors were associated with lower odds of head injury except for dry weather conditions. Crashes that occurred in dry weather conditions were 2.62 times more likely to result in head injury compared to those in wet weather conditions (COR= 2.62, 95% CI: 1.06, 6.478) (Table 5).

**Table 4:** factors associated with head injury in motorcycle crashes- all significant variables

Variable	Cases, n=326 (%)	Controls, n =294 (%)	COR (95% CI)	AOR (95% CI)
**Gender**				
Female	49 (15.03)	80 (27.21)	1	1
Male	277 (84.97)	214 (72.79)	2.11 (1.41,3.15)**	1.75 (1.07, 2.85)*
**Religion**				
Christian	93 (18.37)	54 (28.53)	1	1
Muslim	225 (69.02)	239 (81.29)	0.55 (0.37, 0.803)*	0.64, (0.39, 1.04)
Others	8 (2.45)	2 (0.34)	4.65 (0.57, 38.15)	11.17 (1.12, 111.30)*
**Education**				
No formal education	117 (35.89)	91 (30.95)	1	1
Primary	102 (31.29)	66 (22.45)	1.20 (0.80, 1.82)	1.20 (0.70, 2.08)
Junior high school	25 (7.67)	40 (13.61)	0.49 (0.27, 0.86)*	0.80 (0.49, 1.63)
Senior High/technical school	66 (20.25)	74 (25.17)	0.69 (0.45, 1.07)	1.27 (0.71, 2.28)
Tertiary	16 (4.91)	23 (7.82)	0.54 (0.27, 1.08)	0.90 (0.29, 2.70)
**Occupation**				
Unemployed	90 (27.61)	50 (17.01)	1	1
Formal employment	22 (6.75)	22 (7.48)	0.56 (0.28, 1.10)	0.62 (0.20, 1.93)
Self-employment	177 (54.29)	154 (52.38)	0.64 0.42, 0.96)*	0.78 (0.40, 1.49)
Casual worker	18 (5.52)	29 (9.86)	0.34 (0.17, 0.68)*	0.80 (0.31, 2.07)
Student	19 (5.83)	39 (13.27)	0.27 (0.14, 0.52)**	0.38 (0.16, 0.91)*
**Socioeconomic status**				
Poorest	33 (10.12)	15 (5.10)	1	1
Poor	78 (23.93)	70 (23.81)	0.50 (0.25, 1.01)	0.72 (0.30, 1.72)
Middle	150 (46.01)	152 (51.70)	0.45 (0.23, 0.86)*	0.95 (0.45, 1.50)
Rich	51 (15.64)	41 (13.95)	0.57 (0.27, 1.18)	1.06 (0.31, 2.94)
Richest	14 (4.29)	16 (5.44)	0.40 (0.16, 1.02)	0.83 (0.23, 3.07)
**Collided with**				
Road object	53 (16.26)	31 (10.54)	1	1
Another motorcycle	95 (29.14)	135 (45.92)	0.41 (0.25, 0.69)*	0.76 (0.39, 1.49)
Other vehicle	92 (28.22)	54 (18.37)	0.99 0.57, 1.74)	1.01 (0.49, 2.10)
Pedestrian	86 (26.38)	74 (25.17)	0.68 (0.40, 1.17)	0.81 (0.41, 1.59)
**Riding experience**				
0-5 years	110 (33.74)	107 (36.39)	1	1
6-10 years	131 (40.18)	143 (48.64)	0.89 (0.62, 1.27)	1.32 (0.79, 2.22)
>10 years	85 (26.07)	44 (14.97)	1.88 (1.20, 2.95)*	1.91 (0.97, 3.74)
**Helmet used**				
Used helmet	42 (12.88)	170 (57.82)	1	1
No helmet	284 (87.12)	124 (42.18)	9.27 (6.23, 13.80)**	9.80 (6.22, 15.43)**
**Riding under influence of alcohol**				
Non-alcohol	323 (99.08)	283 (96.26)	1	1
Alcohol use (within 24 hours)	3 (0.92)	11 (3.74)	0.24 (0.07, 0.87)*	0.17 (0.04, 0.70)*

* Significant at p<0.05 level;**significant at p<0.001 level; COR-crude odds ratio; AOR-adjusted odds ratio

**Table 5:** environmental and road conditions associated with head injury in motorcycle crashes

Variable	Cases, n=326(%)	Controls, n =294(%)	COR (95% CI)	AOR (95% CI)
**Weather condition**				
Wet/slippery	7 (2.15)	16 (5.44)	1	1
Dry/dusty	319 (97.85)	278 (94.56)	2.62 (1.06, 6.478)*	1.80 (0.61, 5.34)
**Road type**				
Rural road	57 (17.48)	36 (12.24)	1	1
Urban road	269 (82.52)	258 (87.76)	0.66 (0.42, 1.03)	0.85 (0.45, 1.60)
**Lighting condition**				
Daylight	186 (57.06)	186 (63.27)	1	1
Reduce light/darkness	140 (42.94)	108 (36.73)	1.30 (0.94, 1.79)	1.18 (0.79, 1.76)
**Road surface condition**				
Untarred/earthen road	55 (16.87)	23 (7.82)	1	1
Concrete with potholes	218 (66.87)	186 (63.27)	0.49 (0.29, 0.83)*	0.81 (0.35, 1.89)
Asphalt	53 (16.26)	85 (28.91)	0.26 (0.14, 0.47)**	0.54 (0.22, 1.35)
**Presence of curves**				
No curve	191 (58.59)	114 (38.78)	1	1
Curve	135 (41.41)	180 (61.22)	0.45 (0.32, 0.62)**	0.74 (0.45, 1.22)
**Presence of speed limit**				
No speed limit	98 (30.06)	55 (18.71)	1	1
Speed limit	228 (69.94)	239 (81.29)	0.54 (0.37, 0.78)*	1.55 (0.30, 8.03)
**Visibility of road sign**				
No road sign	108 (33.13)	58 (19.73)	1	1
Road sign	218 (66.87)	236 (80.27)	0.50 (0.34, 0.72)**	0.56 (0.12, 2.69)

* Significant at p<0.05 level; **significant at p<0.001 level; COR-crude odds ratio, AOR-adjusted odds ratio

**Analysis of factors associated with head injuries-multivariable analysis:** Table 4 and Table 5 summarizes the outcomes of multivariable logistic regression analyses examining significant factors of motorcycle-related head injuries. After controlling for all plausible confounders, being male was associated with an increased risk of head injury (AOR=1.75, 95% CI: 1.07, 2.85). The following two factors were associated with decreased risk of head injury: being a student (AOR=0.38, 95% CI: 0.16, 0.91) and alcohol use within 24 hours prior to the crash (AOR=0.17, 95% CI: 0.04, 0.70). Not wearing any helmet was significantly associated with a 10-fold increase in odds of head injury compared to those wearing a helmet (AOR= 9.80, 95% CI: 6.22, 15.43) (Table 4). However, environmental factors assessed in the multivariable model did not reveal any statistically significant associations with a head injury after adjusting for sociodemographic and crash factors (Table 5).

## Discussion

This study sought to identify risk factors for head injuries among survivors of motorcycle crashes in northern Ghana, where motorcycle use has been growing steadily but limited prevention efforts have been implemented. We specifically sought to identify the contributing factors that would be amenable to public health and road safety measures. This study found males, alcohol use within 24 hours, being a student, and non-use of a helmet as the significant independent risk factors. The difference in sociodemographic characteristics such as gender, religion, education, and occupation between the cases and controls in this study is consistent with some, but not all, of the findings from previous studies [5,9,11]. For instance, these studies found that younger drivers have higher crash risks than older drivers, with research indicating that the youngest group of drivers has the highest risk [5,9]. Although the majority of our SMCs were young adults, we found no association between age and head injury. This finding is inconsistent with prior studies on the influence of age and RTI&Ds [5,9]. It was also observed that male victims were more likely to sustain a head injury. The gender of motorcyclists plays an important role in the driver´s crash risk and driving behavior in this effect [15]. Tumwesigye *et al*. [5] reported a lower risk of sustaining motorcycle injuries in Muslims than their counterparts of other religious backgrounds. We included religion in the model to assess the impact of religious practices on patients´ safety orientation. Islam is the predominant religion in northern Ghana. In the bivariate analysis, Muslims were associated with a lower risk of head injury as compared to Christians. The reason for this is not obvious. It might be related to an earlier age of marriage, with the assumption of the role of breadwinner at an earlier age and consequently increased caution. Although it indicates that Muslim riders are likely to adhere to safety practices because of their parenting role [5], the marital status of the patient was not a significant factor for a head injury in this study.

Those without helmets were found to have 10-fold increased odds of head injury. This finding clearly demonstrated that wearing a helmet statistically significantly reduced the risk of head injury and supports existing evidence on the protective effects of helmets in motorcycle crashes [3,9,15-18]. Similar study in Uganda found that those who did not wear helmets at the time of the crash were 2.3 more likely to sustain a head injury [19]. Despite the documented effects of helmets, the rate of helmet use in this study was lower among patients with head injuries. This highlights the importance of helmet promotion campaigns in Ghana, especially in Northern Ghana where the motorcycle is the predominant mode of transportation. Longtime (above 10 years) riding experience was associated with increased odds of head injuries on bivariate, but not multivariable analysis. One would expect that experienced riders know how to maneuver on all kinds of roads and at different times thereby reducing their risk. However, the risk of head injuries increased with an increasing length of riding experience. This finding differs from evidence from studies in other LMICs including Uganda and Thailand [5,18]. These studies reported a protective effect of longtime riding experience. The reason for the difference between those studies and ours is not immediately obvious. Potentially, different sociocultural and behavioral factors may be at play, such as complacency among experienced riders or ingrained habits among experienced drivers, which create barriers to acceptance of recent helmet promotion efforts in Ghana [19]. The current study showed that the effect of alcohol consumption on the odds of head injury is modified by the type of motorcyclist, but an unexpectedly lower risk of head injury with reported use of alcohol was observed in patients with no head injury. This goes against the large body of evidence documenting an increased risk of road traffic crashes with alcohol consumption [5,9,11]. This discordant finding may be due to the fact that the current study used self-report to assess alcohol use. This approach could introduce recall bias with drinkers tending to under-report their alcohol consumption [20]. Weather is a known underlying risk factor of road traffic crashes [21]. The finding of this present study shows that most motorcycle crashes occurred in dry weather, such that survivors of these crashes were twice more likely to suffer head injuries compared to those that occurred in wet weather. The risk of head injuries in the dry season could be explained in different ways. Foremost, the northern regions are desert zones which experience long duration of dry season. Hence, it is likely that most of the crashes occurred in that season. Secondly, there could be a higher risk perception among motorcycle riders during rainy conditions compared with dry conditions.

**Limitation to the study:** a number of limitations were identified; first, the study did not include people who died, and hence we are not able to estimate the effects of the various factors on the risk of fatal injury. Second, the study relied on self-reports by respondents on the injury events, which could not be independently validated. This might be especially a problem for people with head injuries, who might have amnesia for some of the crash events. As this would affect cases more than controls, it could introduce bias into the study. Third, assessment of alcohol consumption was self-reported and information on alcohol audit score or blood alcohol concentration, volumes consumed, and duration of alcohol use before riding were not collected. Fourth, this study did not assess the condition of the motorcycle at the time of the crash. Poor vehicle maintenance, the use of second-hand tires, and braking mechanisms that do not see regular inspection are matters of concern in motorcycle crashes. Finally, driver education and licensure/regularity of licensure renewal were not assessed. Despite these limitations, this study has notable strengths, including having a large sample size and, to our knowledge, being one of the few studies in sub-Saharan Africa to report multivariable-adjusted odds ratios of factors associated with head injuries in motorcycle crashes. Hence, it allows us to make reasonable conclusions about the factors associated with head injuries in an area where motorcycle use is high, but where there have been limited safety interventions yet.

## Conclusion

The study found that being male and not wearing a helmet were associated with increased risk for head injuries in motorcycle crashes in northern Ghana. Being a student and alcohol use within 24 hours were associated with decreased risk. The fact that there were no significant environmental risk factors and few personal or crash characteristics that were significant risk factors indicates that prevention efforts should focus on helmet use. Lack of helmet use was the most significant risk factor in this study. Helmet use is a well-known protective measure in motorcycle crashes. Males were at increased risk of head injury in this study. Hence, emphasis should be placed on this group in helmet promotion efforts. Future studies should measure blood alcohol concentrations of patients and should assess the quality of helmets and the effects of driver education and licensing.

### What is known about this topic

- The risk of sustaining a fatal head injury among motorcyclists has been linked to non-wearing of safety helmets in prior studies;
- The World Health Organization adopted the Haddon matrix model to explore the risk factors of motorcycle injuries into three domains: human, vehicle, and environment;
- There is limited evidence of specific risk factors of head injuries in motorcycle crashes in the Ghanaian context.

### What this study adds

- More than half of survivors of motorcycle crashes suffered head injuries in northern Ghana;
- Predominant head and brain lesions were scalp lacerations, skull fractures, and brain contusion;
- The specific risk factors of head injuries among survivors of motorcycle crashes in northern Ghana included being a male and not wearing a helmet.
